# Photorefractivity and photocurrent dynamics of triphenylamine-based polymer composites

**DOI:** 10.1038/s41598-024-61756-2

**Published:** 2024-05-17

**Authors:** Naoto Tsutsumi, Takafumi Sassa, Tam Van Nguyen, Sho Tsujimura, Giang Ngoc Ha, Yusuke Mizuno, Boaz Jessie Jackin, Kenji Kinashi, Wataru Sakai

**Affiliations:** 1 Faculty of Materials Science and Engineering, Kyoto Institute of Technology, Sakyo, Kyoto 606-8585 Japan; 2 Photonics Control Technology Team, RIKEN Center for Advanced Photonics, Wako, 351-0198 Japan; 3 Department of Materials and Life Science, Graduate School of Science and Technology, Kyoto Institute of Technology, Sakyo, Kyoto 606-8585 Japan; 4 Master’s Program of Innovation Materials, Graduate School of Science and Technology, Kyoto Institute of Technology, Sakyo, Kyoto 606-8585 Japan; 5 Materials Innovation Laboratory, Kyoto Institute of Technology, Sakyo, Kyoto 606-8585 Japan; 6 Present Address: Institute of Applied Science and Technology, Van Lang University, Ho Chi Minh City, Vietnam; 7 Present Address: Faculty of Chemical Technology, Ho Chi Minh City University of Industry and Trade, Ho Chi Minh City, 72000 Vietnam

**Keywords:** Photorefractive properties, Diffraction efficiency, Response time, Two beam coupling, Asymmetric energy transfer, Transient photocurrent, Materials science, Optics and photonics

## Abstract

The photorefractive properties of triphenylamine polymer-based composites with various composition ratios were investigated via optical diffraction, response time, asymmetric energy transfer, and transient photocurrent. The composite consisted of a photoconductive polymer of poly((4-diphenylamino)benzyl acrylate), a photoconductive plasticizer of (4-diphenylamino)phenyl)methanol, a sensitizer of [6,6]-phenyl-C61-butyric acid methyl ester, and a nonlinear optical dye of (4-(azepan-1-yl)-benzylidene)malononitrile. The photorefractive properties and related quantities were dependent on the composition, which was related to the glass transition temperature of the photorefractive polymers. The quantum efficiency (*QE*) of photocarrier generation was evaluated from the initial slope of the transient photocurrent. Transient photocurrents were measured and showed two unique peaks: one in the range of 10^−4^ to 10^−3^ s and the other in the range of 10^−1^ to 1 s. The transient photocurrents was well simulated (or reproduced) by the expanded two-trapping site model with two kinds of photocarrier generation and recombination processes and two different trapping sites. The obtained photorefractive quantity of trap density was significantly related to the photoconductive parameters of *QE*.

## Introduction

First photorefractive (PR) polymer with very low optical diffraction efficiency less than 1% and optical gain less than 0.5 cm^−1^ was reported in1991^1^. Almost full optical diffraction close to 100% including 14% optical loss and optical gain higher than 200 cm^−1^ were reported in PR polymers in 1994^2^. In the past three decades, large number of studies of PR polymers have been dedicated to characterize the photorefractive properties of the optical diffraction efficiency, the optical gain due to the asymmetric energy between two interfered beams, the time response of the optical diffraction, and the trap density. These studies have been summarized in review articles including featured review articles^3–17^.

The photorefractive time response is significantly governed by the space charge field formed by the photorefractive traps captured in the dark region of the photorefractive gratings. In photorefractive polymers, the density of the traps is directly related to the photoconductive properties of the photorefractive polymers. The photoconductive properties are characterized by the quantum efficiency of the photocarrier generation of holes and electrons and the hole mobility in the polymer composites. Commonly, the former is characterized by a xerographic method^18^, and the latter is characterized by a time-of-flight (TOF) method with pulse laser for sample excitation^18^. However, both methods need to be independently established, and occasionally, PR investigators have some difficulties with these methods. For example, PR polymer composites frequently hinder the clear kink observed in the TOF signals due to dispersive carrier conduction, prohibiting the measurement of carrier mobilities.

Alternatively, transient photocurrent measurements with a CW laser are simple and useful tools for investigating the efficiency of the photocarrier generation of holes and electrons, hole mobility, and the trap and detrap properties of the holes in the polymer composites^19,20^. Recently, we investigated the significantly different roles of the photoconductive plasticizer of (2,4,6-trimethylphenyl)diphenylamine (TAA) and (4-diphenylamino)phenyl)methanol (TPAOH) for poly((4-diphenylamino)benzyl acrylate) (PDAA) with (4-(azepan-1-yl)-benzylidene)malononitrile (7-DCST) and^6,6^-phenyl-C61-butyric acid methyl ester (PCBM) prepared with tetrahydrofuran (THF)^21^. In a previous report^22^, the transient photocurrent for the PDAA PR composite with TPAOH photoconductive plasticizer was analysed using a two-trapping site model^19,20^, and that for PDAA PR composite with TAA photoconductive plasticizer was analysed using a single trapping site model^23^. In either model, free positive charge carriers (holes) were photogenerated through photon absorption in a sensitizer molecule, and the generated holes in the transport manifold were recombined with counter sensitizer anions to produce neutral sensitizer molecules^24^ in the recombination process. PCBM is a well-known sensitizer and is assumed to be a PCBM anion in the photoexcitation process, which functions as a recombination site. Furthermore, in the dense solid material of hole transport polymers, PDAA and PCBM are expected to form a charge-transfer (CT) complex, with the functions of a second hole carrier generation site and a second recombination site for the CT anion. Recently, we measured the transient photocurrents with two unique peaks for PDAA/TPAOH/7-DCST/PCBM composites prepared with chloroform. One peak is in the range of 10^−4^ to 10 ^−3^ s and the other peak is in the range of 10^−1^ to 1 s; these peaks cannot be explained using either a single-trapping site model^23^ or a two-trapping site model^19,20^. Here, we propose an expanded two-trapping site model with two kinds of photocarrier generation and recombination processes and two trapping sites. In our strategy, the trap density for the shallow and deep traps for the simulation is equivalent to the photorefractive number density of the trap evaluated from the photorefractive quantities of the space-charge field, trap-limited space-charge field, and phase shift; these quantities are experimentally determined via optical diffraction and optical gain. In the early time region of the transient photocurrent, the rates of trapping and recombination are insignificant^20^; thus, the quantum efficiency for photocarrier generation can be evaluated from the initial slope of the transient photocurrent. The ionization potential and the density of state (DOS) width is estimated using a photoelectron yield spectroscopy (PYS). Particularly, the hole mobility can be evaluated from the DOS width.

In this study, the photorefractive performances of the diffraction efficiency, asymmetric energy transfer, and response time are investigated using four-wave mixing and two-beam coupling methods; additionally, transient photocurrents are simulated using an expanded two-trapping site model for PDAA/TPAOH/7-DCST/PCBM composites prepared with chloroform. The obtained photorefractive quantities of the trap density and the response time of the optical diffraction are comprehensively examined with the photoconductive quantities of the quantum efficiency of the photocarrier generation and the recombination and trapping rates evaluated from the simulation of the transient photocurrent. This fundamental study of photorefractive and photoconductive dynamics of polymer composites will be useful to understand the future study of complex and chaotic signal generation of photorefractive two-wave mixing for the polymer composite which is theoretically shown in photorefractive crystals.

## Materials and measurements

### Materials

A photoconductive polymer of PDAA prepared in our laboratory^25^ was used. A nonlinear optical dye of 7-DCST synthesized in our laboratory^26^ was used. A sensitizer of PCBM supplied from Sigma‒Aldrich Co., USA was used. All other chemicals, unless otherwise stated, were supplied from Wako Pure Chemicals Industries Ltd., Japan.

### Preparation of PR film

PR film consisted of PDAA, TPAOH, 7-DCST, and PCBM was prepared using the same procedure reported in previous paper^27^. The details are shown in previous report^27^.

### Measurement methods

A degenerate four-wave mixing (DFWM) was used to determine the optical diffraction efficiency and response time at 532 nm. Diffraction efficiency was monitored using a weak *p*-polarized probe beam counter-propagated. The detailed measurement procedures were shown in previous paper^27^. The internal diffraction efficiency *η* was calculated as follows:

1 $$ \eta \left( {\text{\% }} \right) = \frac{{I_{{\text{d}}} }}{{I_{{\text{d}}} + I_{{\text{t}}} }} \times 100 $$

where *I*_d_ and *I*_t_ are the intensities of the diffracted and the transmitted probe beams, respectively.

The photorefractive response time *τ* was evaluated by fitting the time dependence of the optical diffraction pattern with the KWW equation:

2 $$\eta = \eta_{0} \left\{ {1 - {\text{exp}}\left[ { - \left( {\frac{t}{\tau }} \right)^{\beta } } \right]} \right\}$$

where *t* is the time, *η*_0_ is the steady-state diffraction efficiency, *τ* is the response time, and *β* is a measure of the dispersion parameter deviating from the single-exponential behaviour (0 <  *β*  ≤ 1).

A two-beam coupling (TBC) with two *p*-polarized interference beams inside the sample device was used to measure the optical gain *Γ* at 532 nm. *Γ* was evaluated using the following equation:

3 $$\Gamma d = \cos \theta_{1} {\text{ln}}\left( {\frac{{I_{1}^{t} \left( {I_{2}^{t} \ne 0} \right)}}{{I_{1}^{t} \left( {I_{2}^{t} = 0} \right)}}} \right) - \cos \theta_{2} {\text{ln}}\left( {\frac{{I_{2}^{t} \left( {I_{1}^{t} \ne 0} \right)}}{{I_{2}^{t} \left( {I_{1}^{t} = 0} \right)}}} \right)$$

where *d* is the thickness of the sample film; *θ*_1_ and *θ*_2_ are the internal refraction angles of beams 1 and 2, respectively; and *I*_1_^t^ and *I*_2_^t^ are the intensities of beams 1 and 2, respectively.

Photoelectron yield spectroscopy (PYS) was performed using the same procedure reported previously with a Bunkoukeiki BIP-KV202GTGK PYS instrument^22^. The composite sample film was spin-coated from chloroform solution. The first derivative of the PYS data was used to determine the density of state (DOS). We measured PYS data three times for each composition.

The absorption coefficient α was determined with a Shimadzu UV-PC2101 UV‒visible spectrophotometer. The glass transition temperature (*T*_g_) was monitored at a heating rate of 10 °C min^−1^ with TA Instruments DSC2920 differential scanning calorimeter.

The transient photocurrent was monitored using a Keithley 6485 picoammeter with a LeCroy 6051A digital oscilloscope for data acquisition; the sample was illuminated at 640 nm using an iFLEX2000 laser with 400 mW cm^−2^ under applying electric field of 40 Vμm^−1^.

## Results and discussion

### Photorefractive properties and evaluation of the trap density

The diffraction efficiency, response time, optical gain, and glass transition temperature measured for PDAA/TPAOH/7-DCST/PCBM with various compositions are summarized in Table 1 with increasing applied electric field, the diffraction efficiency increases, and the response time decreases. For example, higher diffraction efficiencies of 65 to 75%, faster response times of 7 to 12 ms, and higher optical gains of 173 to 193 cm^−1^ are measured at E = 50 V μm^−1^ for the 40/29.4/30/0.6, 35/34.4/30/0.6, and 30/39.4/30/0.6 PDAA/TPAOH/7-DCST/PCBM composites. A relatively lower diffraction efficiency of 14%, lower response time of 94 ms, and lower optical gain of 104 cm^−1^ are measured for the 45/24.4/30/0.6 PDAA/TPAOH/7-DCST/PCBM composite. These differences are strongly related to the glass transition temperature (*T*_g_) of the composite. The former composites have *T*_g_ values between -2 and 0.95 °C which are close to 0 °C, whereas the latter has a *T*_g_ of 11 °C. The difference of the temperature between the *T*_g_ and the measurement temperature in the vicinity of 15 and 20 °C is key. In the former case, molecular motion is allowed, and the orientation of molecules is enhanced because of the rubbery state of the composite due to the temperature difference of 20 °C. However, in the latter case, the molecular motion is partly restricted because of the partial glassy state of the composite due to the small temperature difference.

**Table 1:** *η*, *τ*, and *Γ* measured at E = 45, 50, and 55 V μm^−1^ and *T*_g_ for the sample devices with various compositions.

PDAA/TPAOH/ 7-DCST/PCBM	*E* = 45 V μm^−1^		*E* = 50 V μm^−1^			*E* = 55 V μm^−1^		*T*_g_ (°C)
	*η* (%)	*τ* (ms)	*η* (%)	*τ* (ms)	*Γ* (cm^−1^)	*η* (%)	*τ* (ms)	
45/24.4/30/.6	8	101	14	94	104	18	70	11
40/29.4/30/.6	45	13	75	12	191	84	6	− 2
35/34.4/30/.6	39	8	69	11	173	87	3	0.95
30/39.4/30/.6	47	7	65	7	193	81	4	− 1.67

The photorefractive trap density is an important parameter for evaluating hole trapping events and determining the space charge field for optical diffraction and asymmetric energy transfer.

The number density of trap *T*_i_ is evaluated from *η* and *Γ*. The following is the procedure used to evaluate the photorefractive trap density. Optical diffraction due to the presence of a thick holographic grating in photorefractive polymers is evaluated with a coupled wave theory^28^. The diffraction efficiency *η*_p_ is defined for a *p*-polarized probe beam in a transmission grating as follows:

4 $$\eta_{{\text{p}}} = \sin^{2} \left[ {K{\Delta }n_{{\text{p}}} \cos \left( {\theta_{{\text{B}}} - \theta_{{\text{A}}} } \right)} \right]$$

where $K=\frac{\pi L}{\lambda \sqrt{{\text{cos}}{\theta }_{{\text{A}}}{\text{cos}}{\theta }_{{\text{B}}}}}$, $\Delta {n}_{{\text{p}}}$ is the refractive index modulation, *L* is the sample film thickness, and λ is the wavelength of the probe beam. The optical gain *Γ* is defined as follows:

5 $$\Gamma = \frac{4\pi }{\lambda }\left( {\hat{e}_{1} \cdot \hat{e}_{2} } \right){\Delta }n_{{\text{p}}} \sin {\Phi }$$

where $\Phi$ is the phase shift of the modulated refractive index to the interference illumination pattern and ${\widehat{e}}_{1}$ and ${\widehat{e}}_{2}$ are the polarization unit vectors of the two writing beams^29^. The Kukhtarev model theoretically describes the space charge field^30,31^. The space charge field is related to the phase shift $\Phi$ as follows:

6 $$\tan {\Phi } = \left[ {\frac{{E_{{\text{D}}} }}{{E_{0} }}\left( {1 + \frac{{E_{{\text{D}}} }}{{E_{{\text{q}}} }} + \frac{{E_{0}^{2} }}{{E_{{\text{D}}} E_{{\text{q}}} }}} \right)} \right]$$

where ${E}_{{\text{D}}}$ is the diffusion field, ${E}_{{\text{q}}}$ is the trap-limited space-charge field, and ${E}_{0}$ is the projection of the external electric field onto the grating wave vector ${K}_{{\text{G}}}$. ${E}_{{\text{D}}}$ is defined by ${E}_{{\text{D}}}={K}_{{\text{G}}}kT/e$, where *k*, *T*, and *e* are the Boltzmann’s constant, the absolute temperature, and the electronic charge. If ${E}_{{\text{D}}}$ is dominant, the phase shift is π/2. *T*_i_ is related to the ${E}_{{\text{q}}}$ with the following equation:

7 $$E_{{\text{q}}} = \frac{{eT_{{\text{i}}} }}{{\varepsilon_{0} \varepsilon_{{\text{r}}} K_{{\text{G}}} }}$$

where ${\varepsilon }_{{\text{r}}}$ is the relative dielectric constant of the sample and ${\varepsilon }_{0}$ is the dielectric permittivity in space. The dielectric constant of 3.5 determined before^32^ was used._._ With the measured data of *η*_p_ and *Γ*, $\Phi$ is derived through Eqs. (4) and (5), and then the trap density is finally obtained through Eqs. (6) and (7).

In Kukhtarev model^30,31^, the space-charge field ${E}_{{\text{sc}}}$ is related to ${E}_{{\text{q}}}$, ${E}_{0}$, and ${E}_{{\text{D}}}$, and evaluated using the following equation:

8 $$E_{{{\text{sc}}}} = E_{{\text{q}}} \sqrt {\frac{{E_{{\text{D}}}^{2} + E_{0}^{2} }}{{E_{0}^{2} + \left( {E_{{\text{q}}} + E_{{\text{D}}} } \right)^{2} }}}$$

When ${E}_{{\text{q}}}$ > > ${E}_{0}$, ${E}_{{\text{sc}}} \approx {E}_{0}$ is calculated from Eq. (8), and the small phase shift $\Phi$ is calculated from Eq. (6). In the case of ${E}_{{\text{q}}}$ < < ${E}_{0}$, ${E}_{{\text{sc}}} \approx {E}_{{\text{q}}}$ is calculated from Eq. (8), and $\Phi \approx \pi /2$ is calculated from Eq. (6).

The photorefractive quantities of Φ, *E*_q_, *E*_sc,_ and *T*i determined at E = 50 V μm^−1^ are listed in Table 2.

**Table 2:** Photorefractive quantities of Φ, *E*_q_, *E*_sc,_ and *T*i measured at E = 50 V μm^−1^.

PDAA/TPAOH/7-DCST/PCBM	Φ (°)	*E*_q_ (V μm^-1^)	*E*_sc_ (V μm^-1^)	*T*_i_ (10^16^ cm^-3^)
45/24.4/30/.6	18.5	67	21.2	2.88
40/29.4/30/.6	15.6	81	21.5	3.46
35/34.4/30/.6	12.8	100	21.8	4.27
30/39.4/30/.6	16.5	76	21.5	3.27

### Measurement of the transient photocurrent and expanded two-trapping site model

**Figure 1:**
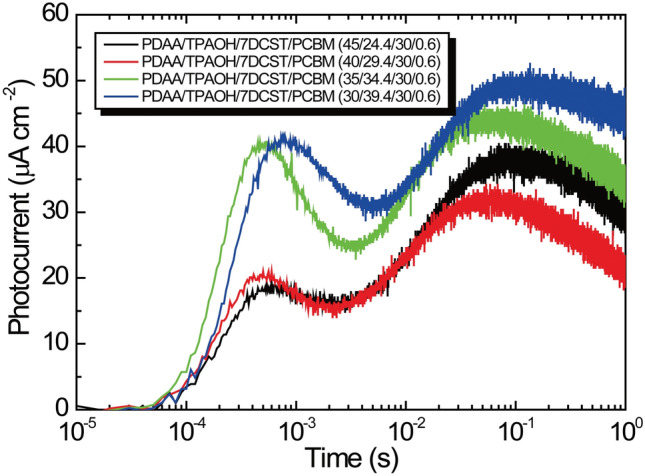
Transient photocurrent profile for the sample with various compositions.

The transient photocurrents measured under illumination with a 400 mW cm^−2^ laser at 640 nm and 40 V μm^−1^ are shown for the sample with various compositions in Fig. 1. These transient photocurrents have two unique peaks; one peak appears at approximately 10^−4^ to 10^−3^ s and another peak appears at 10^−1^ s. A simulation technique with a proper transient photocurrent model is a good way to reproduce these photocurrents and to understand the underlying mechanism. Single-trapping site models^23^ and later two-trapping site models^19,20^ were proposed to reproduce practical transient photocurrents in photorefractive polymers. In the single-trapping site model, the holes are either recombined with sensitizer anions to reproduce the sensitizer molecule, trapped at a single trap site, and then thermally detrapped. In the two-trapping site model, two well-defined (shallow and deep) trapping levels are considered. In a previous report^22^, the transient photocurrent for PDAA PR polymer with TPAOH was analysed using a two-trapping site model, and that for PDAA PR composite with TAA was analysed using a single trapping site model. TAA molecules with HOMO level lower by 0.21 eV than that of host PDAA causes the low scattering effect, which induces the shallow trapping effect in PDAA PR composite with TAA^21^. In this case, compared with faster trapping rate, detrapping rate from shallow trap is much slower^22^. Whereas in PDAA PR composite with TPAOH, HOMO level of TPAOH is slightly higher by 0.05 eV than that of PDAA, which means that TPAOH works as hole manifold in addition to PDAA. PDAA itself works as deep trap sites and the low-scattering effect caused by 7-DCST molecule with HOMO level lower by 0.21 V than that of host PDAA trap site leads to shallow trapping effect^21^. The present transient photocurrents with two unique peaks, as shown in Fig. 1, however, cannot be reproduced by either the conventional single-trapping site model^23^ and the two-trapping site model^19,20^.

**Figure 2:**
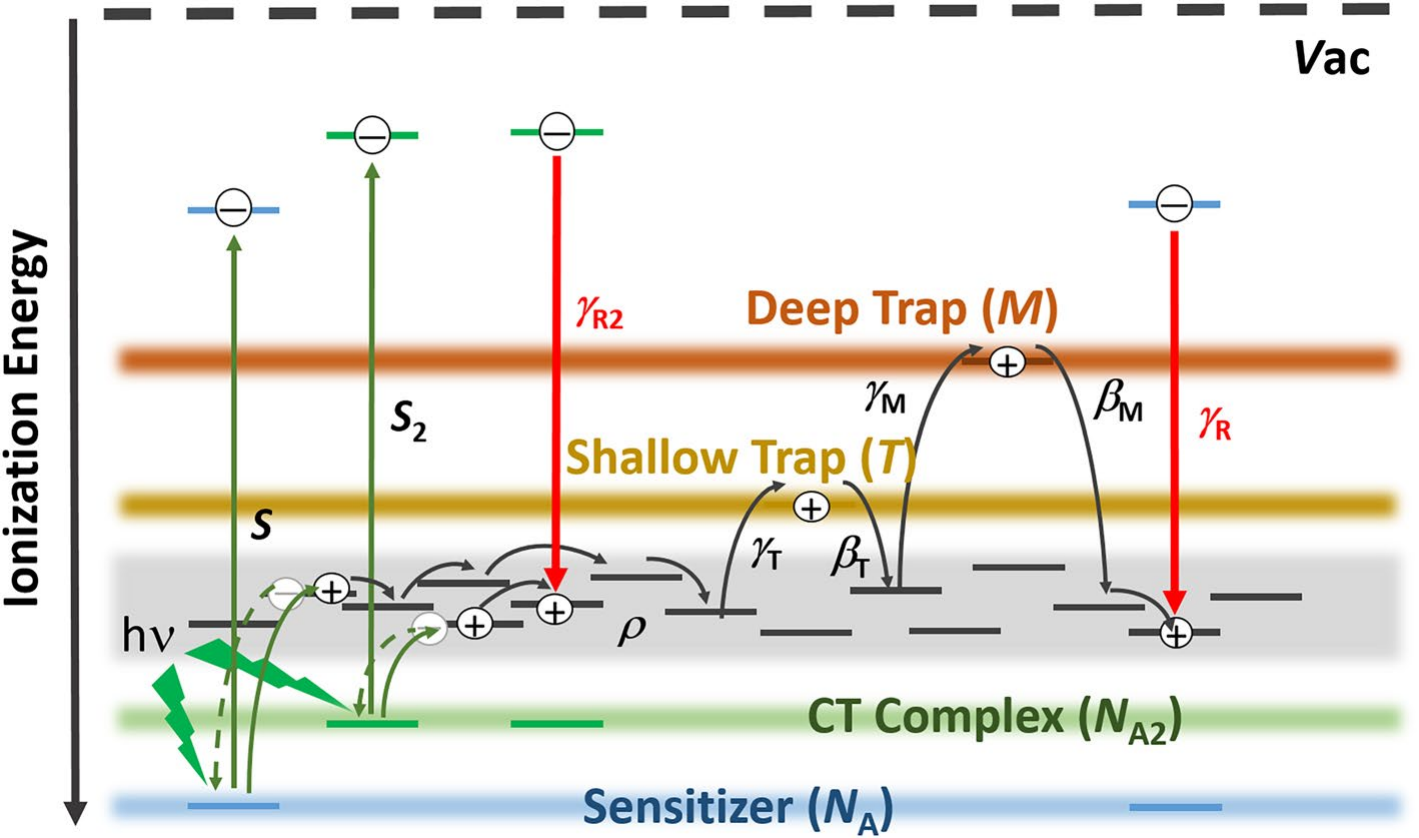
Expanded two-trapping model with two kinds of photocarrier generation and recombination processes.

Here, we speculated that the first peak at approximately 10^−4^ to 10^−3^ s can be reproduced by the conditions in which the trapping event by the shallow trap sites and the recombination of holes nearly occur in the same region. Another peak at approximately 10^−1^ to 1 s is due to the trapping event by the deep trap. Next, we introduce the second photocarrier generation site via the ion pair of the CT complex between the donor and acceptor. This ion pair of the CT complex generates holes and provides the recombination sites for holes. Thus, here, we propose an expanded two-trapping site model with two kinds of photogeneration and recombination processes, as shown in Fig. 2.

In the expanded two-trapping site model, we proposed two kinds of recombination processes: one involves the recombination of holes with a sensitizer anion (PCBM^−^), and the other involves the recombination of holes with a donor and acceptor ion pair site of the CT complex. The expanded two-trapping site model satisfies the following nonlinear equations from (9) to (15) for the photorefractive dynamics:

9 $$J_{{{\text{ph}}}} = e\mu \rho E - eD\frac{\partial \rho }{{\partial x}}$$

10 $$\frac{{{ }\partial \rho }}{\partial t} = \frac{{\partial N_{A}^{ - } }}{\partial t} + \frac{{\partial N_{A2}^{ - } }}{\partial t} - \frac{{\partial T^{ + } }}{\partial t} - \frac{{\partial M^{ + } }}{\partial t} - \frac{1}{e}\frac{{\partial J_{{{\text{ph}}}} }}{\partial x}$$

11 $$\frac{\partial E}{{\partial x}} = \frac{e}{{\varepsilon_{0} \varepsilon_{{\text{r}}} }}\left( {\rho + T^{ + } + M^{ + } - N_{A}^{ - } - N_{A2}^{ - } } \right)$$

12 $$\frac{{\partial T^{ + } }}{\partial t} = \gamma_{{\text{T}}} \left( {T - T^{ + } } \right)\rho - \beta_{{\text{T}}} T^{ + }$$

13 $$\frac{{\partial M^{ + } }}{\partial t} = \gamma_{{\text{M}}} \left( {M - M^{ + } } \right)\rho - \beta_{{\text{M}}} M^{ + }$$

14 $$\frac{{\partial N_{{\text{A}}}^{ - } }}{\partial t} = sI\left( {N_{{\text{A}}} - N_{{\text{A}}}^{ - } } \right) - \gamma_{{\text{R}}} N_{{\text{A}}}^{ - } \rho$$

15 $$\frac{{\partial N_{{{\text{A}}2}}^{ - } }}{\partial t} = s_{2} I\left( {N_{{{\text{A}}2}} - N_{{{\text{A}}2}}^{ - } } \right) - \gamma_{{{\text{R}}2}} N_{{{\text{A}}2}}^{ - } \rho$$

where* J*_ph_ is the current density, *ρ* is the charge carrier density, *μ* is the charge carrier mobility, *E* is the electric field, and *D* is the diffusion coefficient; *e, ε*_0_, and *ε*_r_ are defined above; *N*_A,_ *N*_A2_,* T*, and *M* are the total initial densities of the sensitizers, donor and acceptor pairs of the CT complex, shallow traps, and deep traps, respectively; *N*_A_^−^, *N*_A2_^−^, *T* ^+^, and* M* ^+^ are the densities of the sensitizer anions, donor and acceptor ion pairs, filled shallow traps, and filled deep traps, respectively; *s* and *s*_2_ are the photocarrier generation cross-sections through the sensitizer PCBM and ion pair, respectively; *I* is the light intensity; *γ*_T_ and *γ*_M_ are the shallow and deep trapping rates, respectively;* γ*_R_ and γ_R2_ are the recombination rates by sensitizer anions and ion pairs, respectively; *β*_T_ and *β*_M_ are the detrapping rates from the shallow and deep traps, respectively. The value of *s* for the sensitizer PCBM is given by* s* = *ϕα*λ⁄(*hcN*_A_). Here *α* is the absorption coefficient and *ϕ* is the quantum efficiency (*QE*) for photocarrier generation of PR composite, and *λ*, *c*, and *h* are the wavelength of the light, the speed of light, and the Planck constant, respectively. The value of *s*_2_ for the ion pair is given by* s*_2_ = *ϕ*_2_*α*_2_λ⁄(*hcN*_A2_), where *ϕ*_2_ is the *QE* for photocarrier generation through the ion pair and *α*_2_ is the absorption coefficient of the ion pair.

Conventional two-trapping site model satisfies the nonlinear equations from (9) to (14) and no *N*_A2_ term in Eqs. (10) and (11).

### Photoelectron yield spectroscopy (PYS) and hole mobility

**Figure 3:**
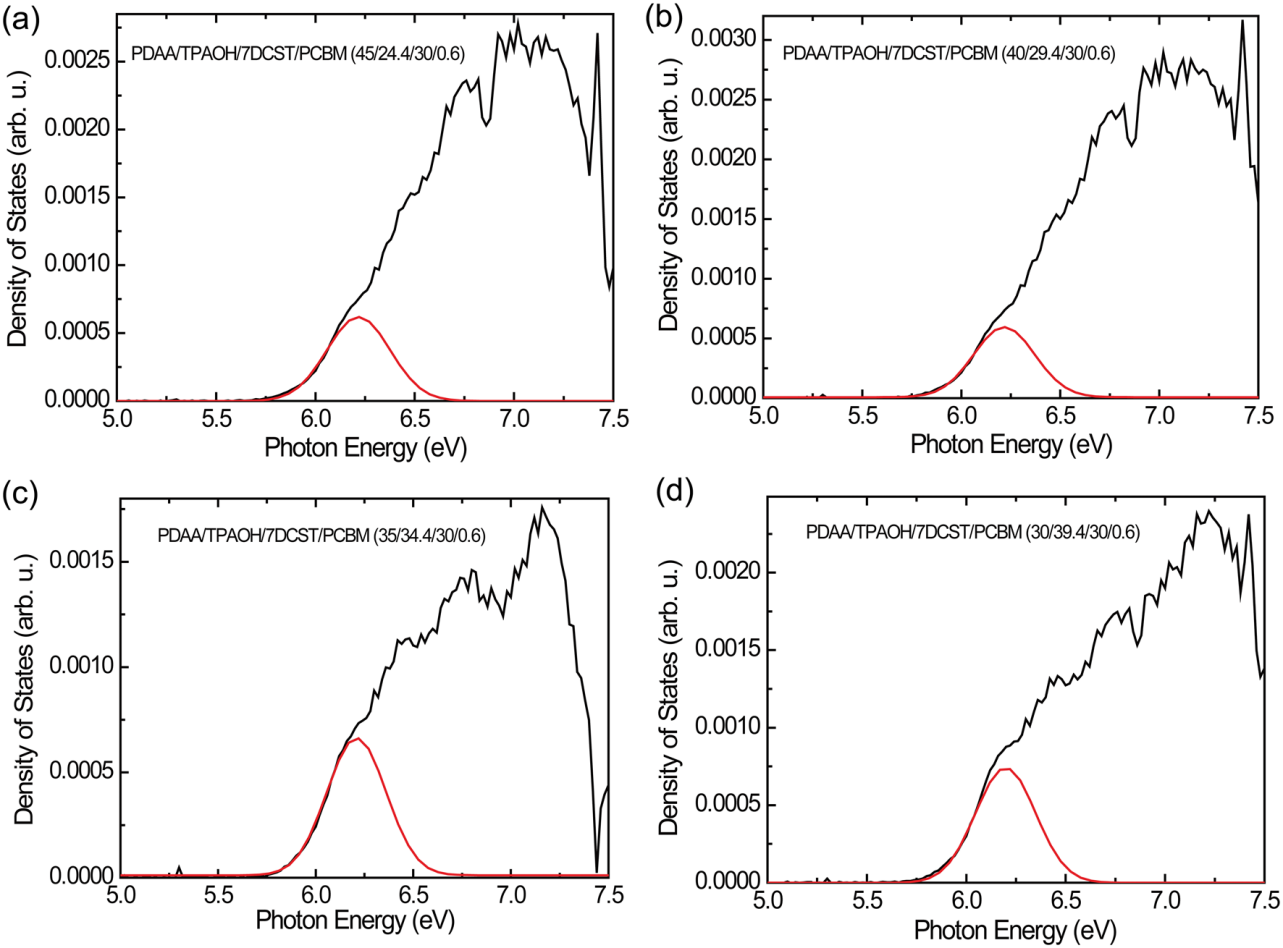
Typical photon energy dependence of DOS curve for PDAA/TPAOH/7DCST/PCBM = 45/24.4/30/0.6 (**a**), 40/29.4/30/0.6 (**b**), 35/34.4/30/0.6 (**c**), and 30/39.4/30/0.6 (**d**). The black curve is the measured DOS profile, and the red curve is the simulated Gaussian curve in the low-photon energy region.

The HOMO levels (ionization potentials) and the width of DOS is usefully estimated using PYS^22^. The HOMO level (ionization potential) of the PR composite is determined by the same procedure reported in previous paper^22^. The DOS spectra are evaluated by differentiating the photoelectron spectra as a function of the photon energy. The energetic disorder in the carrier hopping sites is estimated from the edge part in the DOS spectra at the low-photon energy region. The DOS width was evaluated from the Gaussian peak at the lowest photon energy region using the Gaussian peak separation method in the Origin software. For peak separation, five or more simulations were carried out, and the average DOS width was evaluated. The HOMO level and the DOS width data are summarized in Table 3. The HOMO levels of TPAOH (–5.64 eV) is close to that of PDAA (–5.69 eV)^21^, which indicate that TPAOH sites function as significant hopping sites for the holes in addition to the PDAA hopping sites. This is significantly related to the hole mobility discussed below. Whereas, the HOMO level of the PR composite is in the range between –5.75 and –5.78 eV, which is lower than those of TPAOH and PDAA. The lower HOMO level of PR composite is due to the effect of that of 7-DCST (−5.90 eV)^21^. As listed in Table 3, the DOS width became narrower with increasing TPAOH content. The dipole moment of TPAOH (1.774 D) is smaller than that of PDAA (2.256 D)^32^. Thus, a higher content of TPAOH leads to a lower dipole moment of the PR composite, which directly induces a narrower DOS width. The typical DOS profile for the PR composite is represented by the black curve, and the simulated Gaussian curve at low photon energy is represented by the red curve in Fig. 3.

**Table 3:** HOMO levels, DOS widths, and hole mobilities evaluated.

PDAA/TPAOH/7DCST/PCBM	HOMO (eV)	DOS width (eV)	*μ* (cm^2^ V^−1^ s^−1^)
45/24.4/30/.6	–5.75	0.159	2.49 × 10^–6^
40/29.4/30/.6	–5.77	0.155	3.15 × 10^–6^
35/34.4/30/.6	–5.78	0.152	3.58 × 10^–6^
30/39.4/30/.6	–5.78	0.151	3.76 × 10^–6^

Hole mobility is estimated prior to the transient photocurrent analysis using the simulation techniques. Hole mobility in a dispersive matrix is known to be scattered by the energetic and positional disorder. In the Bässeler’s formalism^33^, considering diagonal and off-diagonal disorder, Monte Carlo simulations lead to the universal law as follows:

16 $$\mu \left( {E,T} \right) = \mu_{0} {\text{exp}}\left[ { - \left( {\frac{2}{3}\frac{\sigma }{kT}} \right)^{2} } \right]{\text{exp}}\left\{ {C\left[ {\left( {\frac{\sigma }{kT}} \right)^{2} - \Sigma^{2} } \right]E^{1/2} } \right\}$$

where *σ* is the standard deviation of Gaussian energy distribution for the hopping site manifold which characterizes diagonal disorder (energetic disorder) and *Σ* is the positional disorder parameter which characterizes off-diagonal disorder, *μ*_0_ is the prefactor mobility, *C* is an empirical constant, and *k*, *T*, and *E* are defined above.

The DOS width can be a measure of diagonal disorder (energetic disorder). Thus, the *σ* value is evaluated from the DOS width. The hole mobility is evaluated using Eq. (16) with a DOS width, *μ*_0_ = 0.01 cm^2^ V^−1^ s^−1^, *C* = 5.30 × 10^−4^ cm^1/2^ V^−1/2^, *Σ* = 3.5, and *E* = 40 Vμm^−1^, and listed in Table 3. These parameters are comparable to those estimated in a previous report^22^. Narrower DOS width reasonably gives higher hole mobility for the sample with lower content of PDAA/higher content of TPAOH. TPAOH itself works as hole transport manifold in addition to PDAA.

### Evaluation of the QE of photocarrier generation

**Figure 4:**
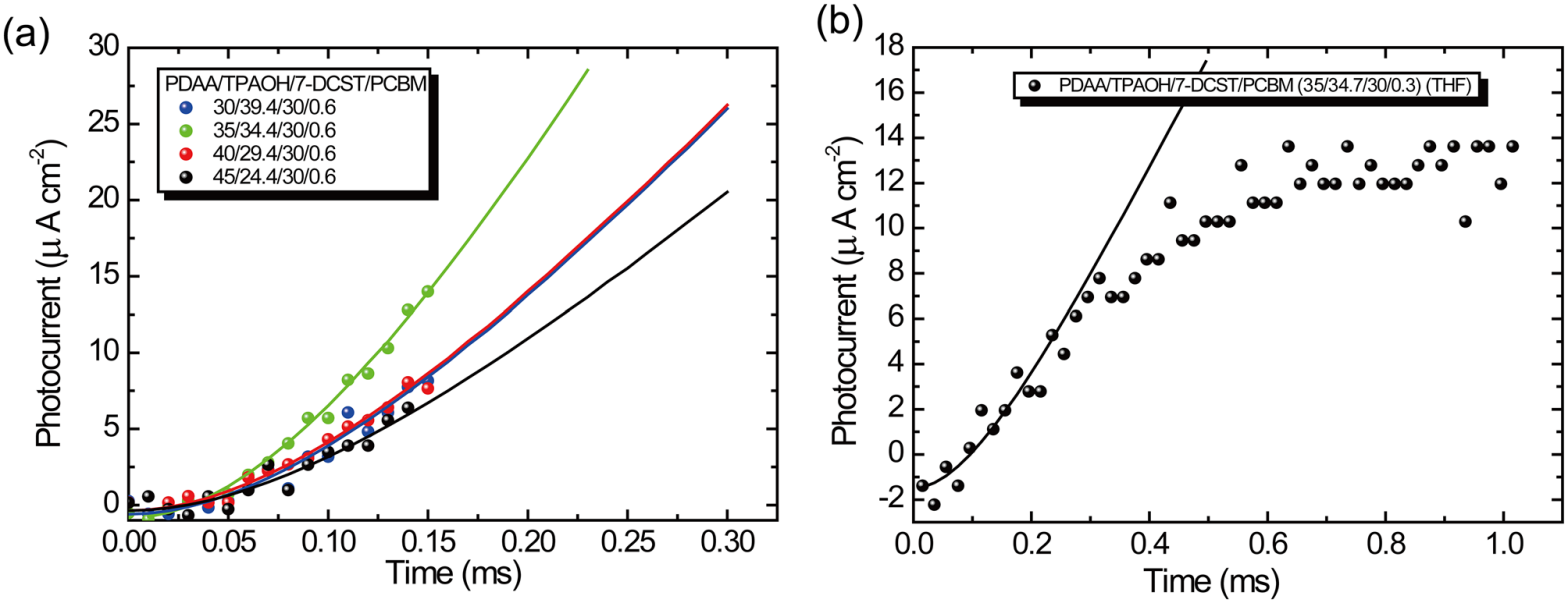
(**a**) Photocurrent in the initial time region for the composite with various compositions. The fitted parameters of *dj*_photo_/*dt* are listed in Table 4 for each composite. (**b**) Photocurrent in the initial time region for the composite prepared with a THF-cast solvent. The fitted parameter *dj*_photo_/*dt* is 0.05 A cm^−2^ s^−1^. The solid curves are fitted using Eq. (18).

The quantum efficiency for photocarrier generation should also be evaluated prior to the transient photocurrent analysis. In our previous report^22^, the *QE* for photocarrier generation was evaluated from the response time^34^. However, for the present case, the response time for PDAA/TPAOH/7-DCST/PCBM (45/24.4/30/0.6) is significantly affected by the NLO chromophore orientation in the glass transition region discussed above. Thus, the evaluation of *ϕ* using photorefractive response time is not suitable for the present PR composites. Alternatively, the *QE* of photocarrier generation *ϕ* can be evaluated from the initial slope of the transient photocurrent $\frac{d{j}_{{\text{photo}}}}{dt}{|}_{t=0}$ as follows:

17 $$\frac{{dj_{{{\text{photo}}}} }}{dt}|_{t = 0} = \mu eE\frac{\phi \alpha \lambda }{{hc}}I$$

where *μ*, *e*, *E*, *ϕ*, *α*, *λ*, *h*, *c*, and *I* are defined above^20^. In the early time region of the transient photocurrent, the rates of trapping and recombination are insignificant^20^; thus, *ϕ* can be evaluated with *μ* determined above and is listed in Table 3. The initial transient photocurrents measured under illumination with a 400 mW cm^-2^ laser at 640 nm and 40 V μm^-1^ are shown for PDAA/TPAOH/7-DCST/PCBM with various compositions in Fig. 4 (a). For the present measurement system, the picoammeter has an electronic time constant *τ*_RC_ = 120 μs, the measured initial response current *j*_τ_ is affected by this time constant, and *j*_τ_ is described as follows:

18 $$j_{{\uptau }} \left( t \right) = \frac{{dj_{{{\text{photo}}}} }}{dt}|_{t = 0} \left( {t - \tau_{{{\text{RC}}}} \left[ {1 - e^{{ - \frac{t}{{\tau_{{{\text{RC}}}} }}}} } \right]} \right)$$

The measured initial photocurrent *j*_τ_ is fitted using Eq. (18), and $\frac{d{j}_{{\text{photo}}}}{dt}{|}_{t=0}$ is evaluated and listed in Table 4.

**Table 4:** The initial slope of the photocurrent (*dj*_photo_/*dt*), the absorption coefficient measured at 640 nm (*α*_640_), and the quantum efficiency of photocarrier generation were evaluated.

Composition	*dj*_photo_/*dt* (A cm^−2^ s^−1^)	*ϕα* + *ϕ*_2_*α*_2_ (cm^−1^)	*α*_640_ (cm^−1^)	*α* (cm^−1^)	*α*_2_ (cm^−1^)	*ϕ* (10^−2^)	*ϕ*_2_ (10^−2^)
45/24.4/30/.6	0.11	0.535	22	2.5	19.5	4.45	2.17
40/29.4/30/.6	0.14	0.538	12.6		10.1		4.23
35/34.4/30/.6	0.23	0.779	10.8		8.3		8.05
30/39.4/30/.6	0.14	0.451	10.8		8.3		4.09

### Analysis of the transient photocurrent

**Figure 5:**
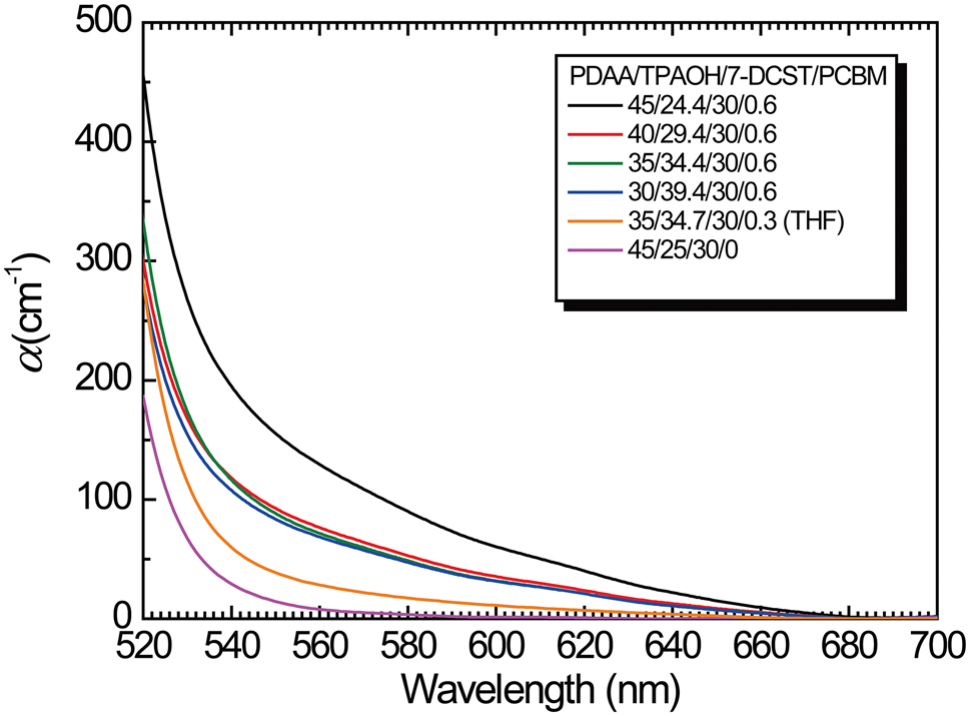
Absorption spectra for various compositions of the PDAA/TPAOH/7-DCST/PCBM composites.

*T*_i_ values listed in Table 2, and *μ* values listed in Table 3 are used as fixed parameter values for the analysis of the transient photocurrent. In our proposed model, two types of photocarrier generation sites are considered. Figure 5 shows the absorption spectra. The introduction of PCBM results in a large absorption in the entire wavelength region. This absorption is caused by the CT complex between PDAA and PCBM in addition to the PCBM sensitizer only. Furthermore, for the sample without PCBM, no absorption (see purple curve in Fig. 5) appears in the 640 nm wavelength region, and the possibility of the CT complex of PDAA and 7-DCST is negligibly small. Thus, only the PCBM sensitizer and CT complex between PDAA and PCBM are photocarrier generation sites.

From the materials’ point of view, interesting results for the solubility of PCBM in solvent have been reported; the solubility of PCBM in chloroform is much greater than that in THF^35^. This finding suggests that it is easier to form CT complexes in chloroform than in THF. Therefore, we postulate that the photocarrier generated for the sample prepared with THF is only PCBM and not the CT complex, whereas that for the sample prepared with chloroform consists of both PCBM and the CT complex. For the simulation analysis, we speculate that the initial slope for the sample prepared with THF is only caused by the photocarrier generation through PCBM sensitizer. Thus the initial slope of the transient photocurrent for the sample PDAA/TPAOH/7-DCST/PCBM (35/34.7/30/0.3) with smaller PCBM of 0.3 wt% prepared with THF was measured and the product of *ϕα* = 0.169 cm^−1^ is evaluated from the initial slope of *dj*_photo_/*dt* = 0.05 A cm^−2^ s^−1^ using Eq. (17), which is shown as solid line in Fig. 4 (b). The absorption spectrum of this sample is shown in Fig. 5 and *α* value of 3.8 cm^−1^ is measured at 640 nm for the sample prepared with THF. The *α* value of 3.8 cm^−1^is reasonable and close to that of 3.05 cm^−1^ estimated in polycarbonate film^36^. Then *ϕ* = 0.0445 is evaluated for *QE* of only PCBM as listed in Table 4. The simulated transient photocurrent for PDAA/TPAOH/7-DCST/PCBM (35/34.7/30/0.3) (THF) using a conventional two-trapping model is shown in Fig. S1 with fitting parameters summarized in Tables S1 and S2 in Supplementary Information.

Next, for the analysis of the sample prepared with chloroform, the term *ϕα* + *ϕ*_2_*α*_2_ is used instead of the term *ϕα* in Eq. (17), and the values evaluated are listed in Table 4. Unfortunately, *ϕα* obtained from the sample with THF (0.169 cm^−1^) does not fully reproduce the first peak in the vicinity of 0.1 to 1 ms. The simulation using a value of *ϕα* smaller than 0.169 cm^−1^ provides a better fit and can reproduce the first peak, as shown in Fig. S2 in the Supplementary Information. In the sample prepared with chloroform, the *α* value at 640 nm includes the contribution of CT complex between PDAA and PCBM in addition to the only PCBM, causing the reduction of *α* and *ϕα*. The best fit is given by *ϕα* = 0.112 cm^−1^.Thus *α* vale of 2.5 cm^−1^ is evaluated as PCBM absorption for the sample prepared with chloroform. The absorption coefficient of the CT complex *α*_2_ is *α*_2_ = *α*_640_ − *α*, and the results are listed in Table 4. The value of *α*_2_ increases with increasing PDAA content; this is reasonable because the interaction between PDAA and PCBM is a function of PDAA content and becomes stronger with increasing PDAA content. The obtained *ϕ* and *ϕ*_2_ are listed in Table 4. An *N*_A_ of 4.76 × 10^18^ cm^−3^ is experimentally determined as a fixed parameter. On the other hand, since a small amount of PCBM forms a CT complex with the donor PDAA, the value of *N*_A2_ is estimated as follows: *N*_A2_ = 2 × 10^−4^ × *N*_A_ = 9.52 × 10^−14^ cm^−3^. The reason for determing *N*_A2_ value is summarized in Supplementary Information.

**Figure 6:**
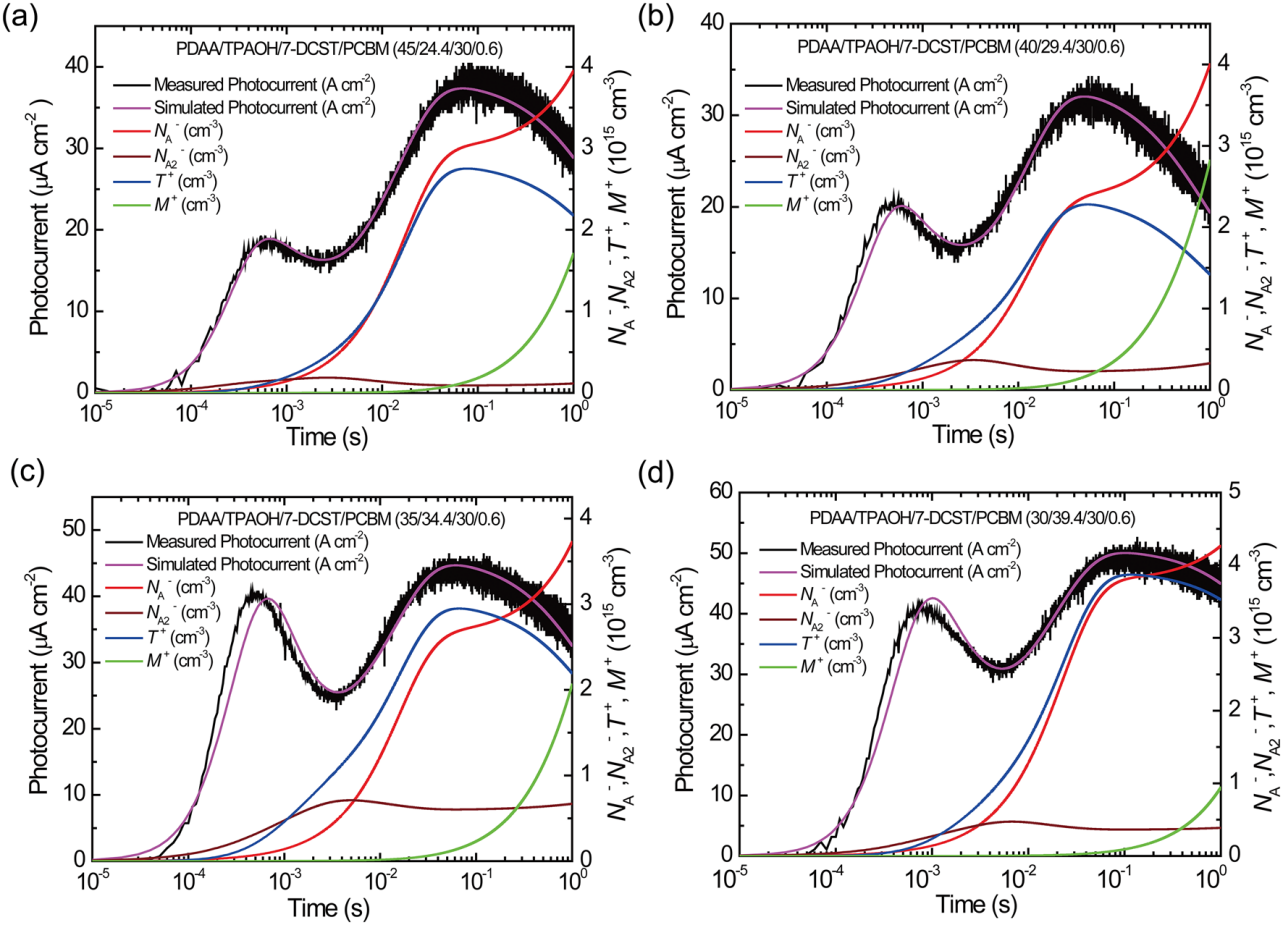
Simulation of the transient photocurrent for PDAA/TPAOH/7-DCST/PCBM with composition ratios of (**a**) 45/24.4/30/0.6, (**b**) 40/29.4/30/0.6, (**c**) 35/34.4/30/0.6, and (d) 30/39.4/30/0.6. Black plots, measured transient photocurrent; pale purple curve, simulated transient photocurrent; red curve and red wine colour curve, transient density for the sensitizer anion *N*_A_^−^ and transient density of the ion pair *N*_A2_^−^, respectively; blue curve and green curve, transient density for filled shallow traps *T*^+^ and transient density for filled deep traps *M*^+^, respectively.

Figure 6 shows the transient photocurrent fitted by the expanded two-tapping site model with the parameters listed in Table 5. The present transient photocurrents with two peaks are effectively simulated by the expanded two-trapping site model. As listed in Table 5, the recombination rate of *γ*_R2_ is one or two orders of magnitude higher than that of *γ*_R_.

**Table 5:** Summary of photoconductive parameters for simulating transient photocurrent.

Composition	*γ*_R_ (cm^3^ s^−1^)	*γ*_R2_ (cm^3^ s^−1^)	*γ*_T_ (cm^3^ s^−1^)	*T, M* (cm^−3^)	*β*_T_ (s^−1^)	*γ*_M_ (cm^3^ s^−1^)	*β*_M_ (s^−1^)
45/24.4/30/.6	2.0 × 10^–13^	2.3 × 10^–11^	7.0 × 10^–14^	2.88 × 10^16^	155	3.0 × 10^−16^	0.01
40/29.4/30/.6	3.7 × 10^–13^	1.15 × 10^–11^	1.2 × 10^–13^	3.46 × 10^16^	270	7.0 × 10^−16^	0.01
35/34.4/30/.6	2.7 × 10^–13^	2.7 × 10^–12^	8.0 × 10^–14^	4.27 × 10^16^	210	3.0 × 10^−16^	0.01
30/39.4/30/.6	1.8 × 10^–13^	3.6 × 10^–12^	5.5 × 10^–14^	3.27 × 10^16^	85	1.5 × 10^−16^	0.01

*N*_A_ = 4.76 ×  10^18^ cm^−3^, *N*_A2_ = 2 × 10^−4^ × *N*_A_ = 9.52 × 10^14^ cm^−3^.

As shown in Fig. 6, the transient density of the photogenerated *N*_A2_^−^ (red wine colour curve in the figure) and that for filled shallow traps *T*^+^ (blue curve in the figure) mainly contribute to the first peak in the time region between 10^−4^ and 10^−3^ s. The large depression of the transient photocurrent in the time region above 10^–1^ s is due to the large increase in transient density for filled deep traps *M*^+^ (green curve in the figure). However, strictly speaking, the first peak in the time region between 10^−4^ and 10^−3^ s is reproduced at later time region as shown in Fig. 6b–d. This may be because of under estimation of *ϕ*_2_ of CT complex between PCBM and PDAA. Namely, the initial slope of the transient photocurrent would be reduced by faster recombination of the generated carriers and under estimated. As a reference, well-fitted result is presented for PDAA/TPAOH/7-DCST/PCBM (30/39.4/30/0.6) in Fig. S3 in Supplementary Information.

### Comparison of photorefractive and photocurrent quantities

**Figure 7:**
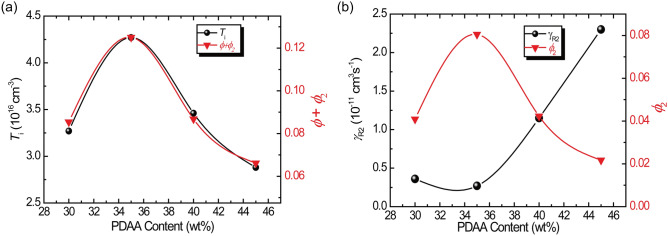
(**a**) PDAA content dependence of *T*_i_ and *ϕ* + *ϕ*_2_. (**b**) PDAA content dependence of *γ*_R2_ and *ϕ*_2_. The solid line is a guide to the eye.

Here, let us consider the dependence of *ϕ* + *ϕ*_2_ and *T*_i_ on the PDAA content. The PDAA content dependence of both *ϕ* + *ϕ*_2_ and *T*_i_ are shown in Fig. 7a. The values of *ϕ* + *ϕ*_2_ and *T*_i_ peak at a PDAA content of 35 wt%, and the dependence of *T*_i_ on the PDAA content effectively follows that of *ϕ* + *ϕ*_2_. This result is interesting because almost all the photogenerated charge carriers are effectively trapped by trap sites to form a space charge field. The peak of *QE* of photocarier generation at PDAA content of 35 wt% is explained by the PDAA content dependence of the recombination coefficient *γ*_R2_ as shown in Fig. 7b. Higher recombination coefficient, i.e., recombination rate, with increasing PDAA content is significantly related to the larger depression of the photocarrier generation^38^.

The question is arised why the optical gain Γ and hole mobility are the highest for the 30/39.4/30/0.6 PDAA/TPAOH/7-DCST/PCBM composites, but the number density of the traps Ti and the QE for photocarrier generation through the ion pair are the highest for the 35/34.4/30/0.6 PDAA/TPAOH/7-DCST/PCBM composites? One reason is that the maximum charge quantum efficiency is not necessarily the case when the mobility is maximum. For example, quantum efficiency varies greatly depending on the size of the HOMO gap between the charge-generating molecule and the charge-transporting molecule.

**Figure 8:**
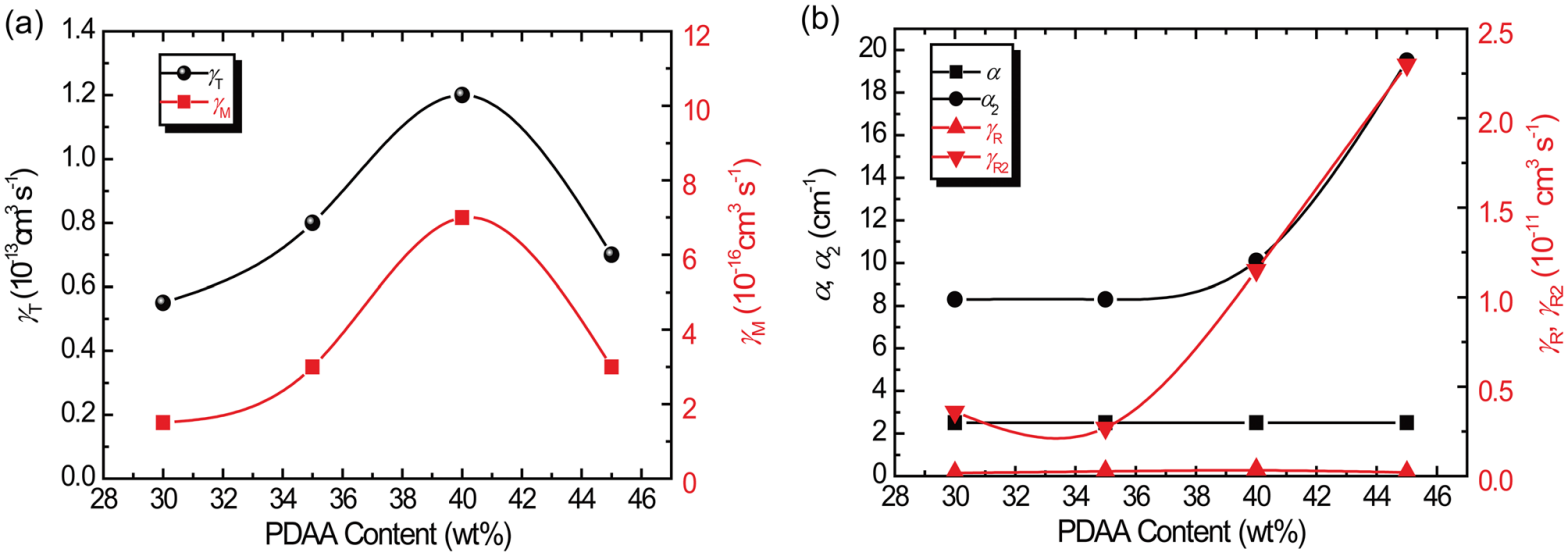
(**a**) PDAA content dependence of *γ*_T_ and *γ*_M_. (**b**) PDAA content dependence of *γ*_R_, *γ*_R2_,*α*, and *α*_2_. The solid line is a guide to the eye.

The trapping rates for shallow and deep traps evaluated from the analysis of transient photocurrent are plotted as a function of PDAA content in Fig. 8. The trapping rates for the shallow traps, *γ*_T,_ and for the deep traps, *γ*_M_, reaches a maximum at a PDAA content of 40 wt%. Each recombination rate mainly follows the corresponding absorption coefficient. Thus, the strong absorption likely leads to significant recombination.

The trapping time can be defined by the inverse of the trapping rate and is listed in Table 6. For comparison, the photorefractive response times are also listed in Table 6. The trapping time for the shallow trap is on the order of sub-millisecond, whereas that for deep trap is on the order of sub-second, and the photorefractive response time is between them.

**Table 6:** Trapping rates *γ* × *T* and times (*γ* × *T*)^−1^ for the shallow and deep traps, as well as the photorefractive response time.

Composition	Trapping rate (s^-1^) *γ*_T_ × *T γ*_M_ × *M*		Trapping time (ms) (*γ*_T_ × *T*)^−1^ (*γ*_M_ × *M*)^−1^		Photorefractive response time (ms)
45/24.4/30/.6	2016	8.64	0.50	116	94
40/29.4/30/.6	4152	24.2	0.24	41.3	12
35/34.4/30/.6	3416	12.8	0.29	78.1	11
30/39.4/30/.6	1799	4.91	0.56	204	7

Using the same procedure previously reported^22^, the trap state (in eV) Δ*E* is evaluated from the inverse *β*^37^ as follows:

19 $$t_{{{\text{tr}}}} = \frac{a}{v}{\text{exp}}\left( {\frac{\Delta E}{{kT}}} \right) = \frac{1}{\beta }$$

where *k* and *T* are defined above, *t*_tr_ is the trap residing time, *v* is the velocity for hopping, and *a* is the average distance of hopping. *v* is calculated from the hole mobility *μ* and *E* with *μ* = *v*/*E*. The detrapping rate, time and Δ*E* are summarized in Table 7. Δ*E* for the shallow is between 0.28 and 0.32 eV, and that for the deep trap is between 0.53 and 0.55 eV. These values are reasonable for the trap state.

**Table 7:** Summary of the detrapping rate *β* and time *β* ^−1^, as well as the value for trap state Δ*E* for shallow and deep traps.

Composition	Detrapping rate (s^−1^) *β*_T_ *β*_M_		Detrapping time (ms) *β*_T_^*−*1^ *β*_M_^−1^		Shallow trap Δ*E* (eV)	Deep trap Δ*E* (eV)
45/24.4/30/.6	155	0.01	6.45	100,000	0.288	0.536
40/29.4/30/.6	270	0.01	3.7	100,000	0.280	0.542
35/34.4/30/.6	210	0.01	4.8	100,000	0.290	0.546
30/39.4/30/.6	85	0.01	11.8	100,000	0.315	0.547

## Conclusions and perspectives

The photorefractive and photoconductive dynamics of the PDAA/TPAOH/7-DCST/PCBM composite were investigated. The photorefractive properties and related quantities are dependent on the composition and related to the *T*_g_ of the composite. High diffraction efficiencies more than 60%, fast response times around ca. 10 ms, and high optical gains close to 200 cm^−1^ are measured for 40/29.4/30/0.6, 35/34.4/30/0.6, and 30/39.4/30/0.6 PDAA/TPAOH/7-DCST/PCBM composites with *T*_g_ close to 0 °C. Whereas, a lower diffraction efficiency less than 15%, a slower response time of 94 ms, and a relatively lower optical gain of 104 cm^−1^ are measured for the PDAA/TPAOH/7-DCST/PCBM composite at 45/24.4/30/0.6 with *T*_g_ closer to room temperature. These differences are significantly related to the *T*_g_ of the composite and the temperature difference between the *T*_g_ and the measured temperature.

Transient photocurrents of two peaks are measured; one peak is in the range of 10^−4^ to 10^−3^ s, and the other peak is in the range of 10^−1^ to 1 s. To reproduce the unique transient photocurrent, we propose an expanded two-trapping model with two kinds of photocarrier generation and recombination processes. One photocarrier generation site is PCBM only and the other is CT complex between PCBM and PDAA. CT complex between PCBM and PDAA with very small amount compared to PCBM plays an important role for both photocarrier generation and recombination sites to produce peak in the range of 10^−4^ to 10^−3^ s. With proper parameters of the photorefractive number density of the traps and the quantum efficiency for photocarrier generation, we can properly reproduce the measured transient photocurrent. The first peak at approximately 10^−4^ to 10^−3^ s can be reproduced under the following the condition: the trapping event by the shallow trap sites and the recombination of holes occur in almost the same time region. The other peak at approximately 10^−1^ to 1 s is caused by the trapping event by the deep trap.

The density of the photorefractively trapped *T*_i_ increases with increasing PDAA content from 30 to 35 wt%, reaches a maximum at PDAA/TPAOH/7-DCST/PCBM (35/34.4/30/0.6) and decreases with increasing PDAA content from 35 to 45 wt%. The PDAA content dependence of *T*_i_ is effectively characterized by the quantum efficiency of photocarrier generation.

This study showed that the difference of *T*_g_ due to the different PR composition significantly affected the PR performances. However, in the present report the effect of temperature and *T*g on the transient photocurrent has not been investigated, which will be figured out by our future work.

### Supplementary Information

Supplementary Information.

## Data Availability

The datasets used and/or analysed during the current study available from the corresponding author on reasonable request.
