# Supplementary material for: Photorefractivity and photocurrent dynamics of triphenylamine-based polymer composites
Source: Sci Rep. 2024 May 17;14:11286. doi: 10.1038/s41598-024-61756-2 (PMC11101462; doi:10.1038/s41598-024-61756-2)
Supplement: Supplementary file 1 — Supplementary Information. [file 41598_2024_61756_MOESM1_ESM.docx]

**Supplementary Information**

*Determination of QE of Photocarrier Generation φ*

The transient photocurrent for the sample of PDAA/TPAOH/7-DCST/PCBM (35/34.7/30/0.3) prepared with THF was simulated using a conventional two-trapping model with *φ* =0.0445, *α*  cm^−1^ listed in **Table S1**, and trapping and recombination parameters listed in **Table S2**. Measured photocurrent is well reproduced by a conventional two-trapping model with single photocarrier generation and recombination process. Thus we evaluated that the *QE* of photocarrier generation of only PCBM is *φ* =0.0445.


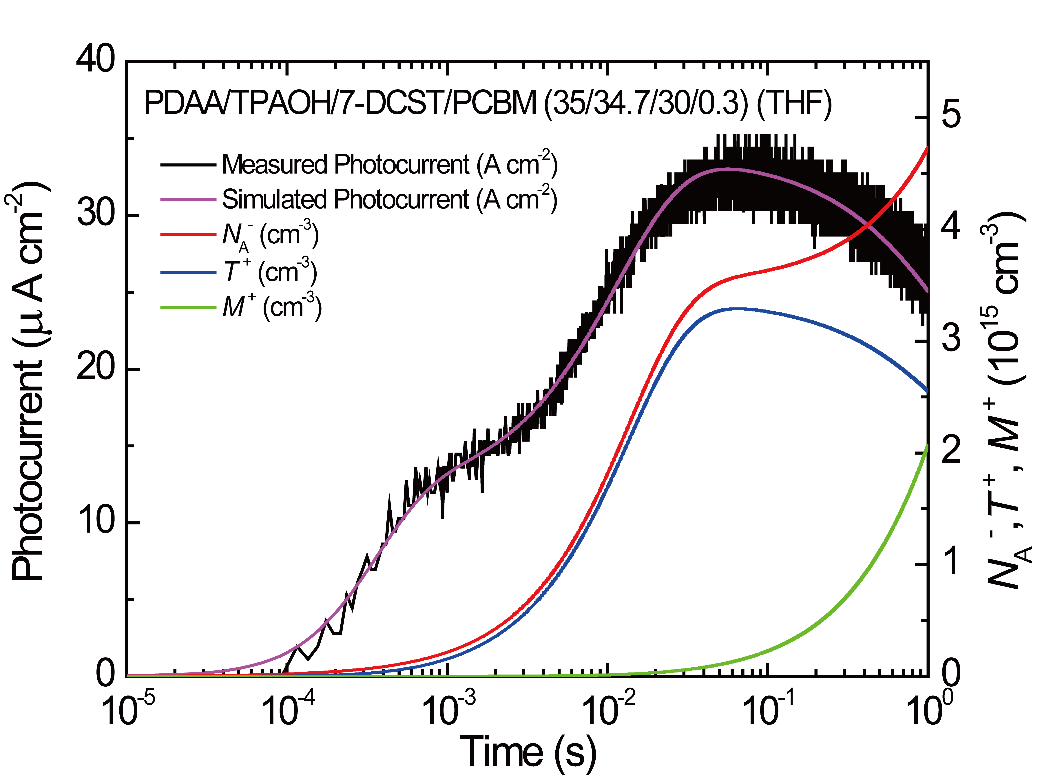


**Figure S1**. Simulation of the transient photocurrent for PDAA/TPAOH/7-DCST/PCBM (35/34.7/30/0.3) prepared with THF using a conventional two-trapping model with *φ* =0.0445, *α*  cm^−1^, and trapping and recombination parameters listed in **Table S2**.

**Table S1**. The initial slope of the photocurrent (*dj*_photo_/*dt*), the absorption coefficient measured at 640 nm (*α*_640_), and the quantum efficiency of photocarrier generation were evaluated.

| composition | *dj*_photo_/*dt* (A cm^−2^s^−1^) | *φα * (cm^−1^) | *α*_640_ (cm^-1^) | *α* (cm^-1^) | *φ* (10^−2^) |
| --- | --- | --- | --- | --- | --- |
| 35/34.7/30/.3 (THF) | 0.05 | 0.169 | 3.8 | 3.8 | 4.45 |

**Table S2.** Summary of photoconductive parameters for simulating transient photocurrent.

| composition | *γ*_R_ (cm^3^ s^-1^) | *γ*_T_ (cm^3^ s^-1^) | *T, M* (cm^-3^) | *β*_T_ (s^-1^) | *γ*_M_ (cm^3^ s^-1^) | *β*_M_ (s^-1^) |
| --- | --- | --- | --- | --- | --- | --- |
| 35/34.7/30/.3 (THF) | 4.2×10^-13^ | 9.0 ×10^-14^ | 4.27 ×10^16^ | 155 | 4.0×10^−16^ | 0.01 |

*Determination of proper product of φ and α*

The product of *φ* and *α* is varied to reproduce the first peak in the vicinity of 1 ms. The *φ* and *α* product of 0.169 cm^−1^ cannot be effectively reproduced for the first peak as shown in **Figure S2**. A value less than 0.330 cm^−1^ improved the fitting. The *φ* and *α* product of 0.112 cm^−1^ or less provides a better fit. We determined *φ* and *α* product of 0.112 cm^−1^.


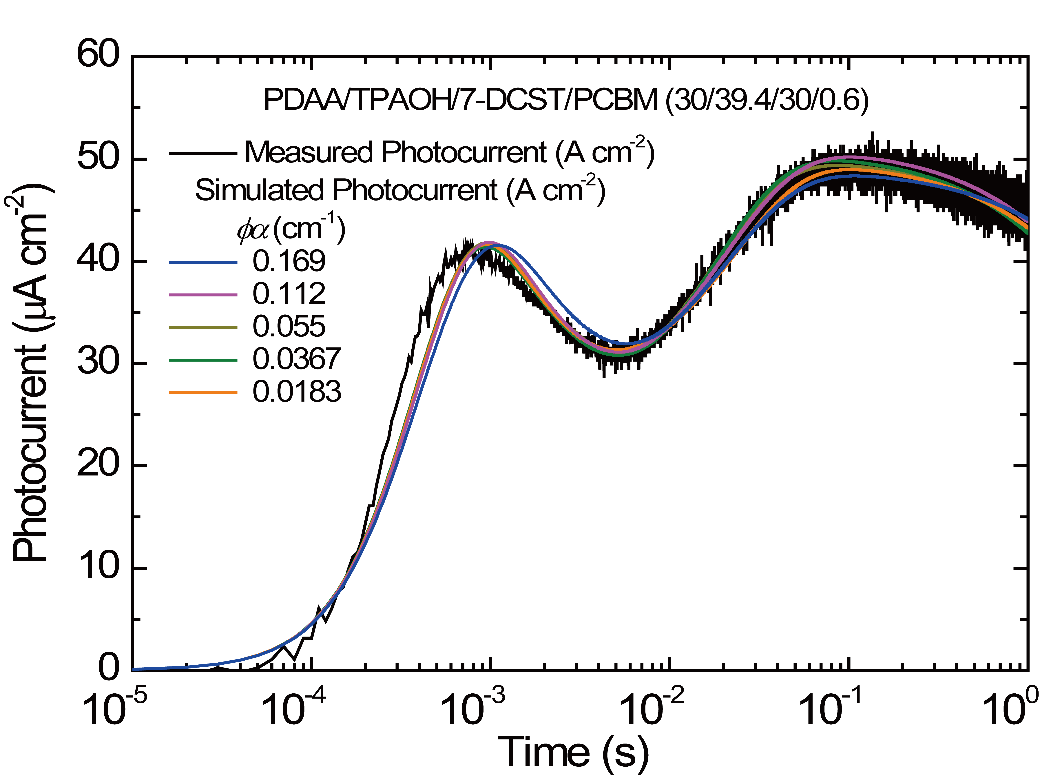


**Figure S2**. Simulation of the transient photocurrent with varying the product of *φ* and *α* for PDAA/TPAOH/7-DCST/PCBM (30/39.4/30/0.6).

*Determination of N_A2_ Value*

In the present system, we thought that the number density of CT complex should be very small, because the absorption spectrum of CT complex is not clearly observed. In the simulation process, changing the number density of *N*_A2_ from equivalent to *N*_A_ to 1×10^−3^×*N*_A_ does not give us any major change in first peak, and when decreasing *N*_A2_ value from 1×10^−3^×*N*_A_ first peak starts to gradually decrease and at *N*_A2_ value lower than 2×10^−5^×*N*_A_ first peak is disappeared and cannot be reproduced. So we picked up *N*_A2_ value of 2×10^−4^×*N*_A_ between them.

*Best Fitting of Transient Photocurrent*

For PDAA/TPAOH/7-DCST/PCBM (30/39.4/30/0.6), *dj*_photo_/*dt* = 0.14 A cm^−2^s^−1^ was measured from the initial slope of the transient photocurrent, but this value would be under estimated by the faster recombination of the photocarrier generated. However, we could not compensate this problem in the calculation process. So, as a reference, we postulated *dj*_photo_/*dt* = 0.165 A cm^−2^s^−1^ and evaluated *φ*_2_ of 5.06 × 10^−2^ as listed in **Table S3**. Transient photocurrent was simulated using this parameter. Parameters are summarized in **Tables S3** and **S4**. Well-fitted transient photocurrent is shown for PDAA/TPAOH/7-DCST/PCBM (30/39.4/30/0.6) in **Figure S3**.

**Table S3**. Summary of the parameters for better-fitting for PDAA/TPAOH/7-DCST/PCBM (30/39.4/30/0.6): the initial slope of the photocurrent (*dj*_photo_/*dt*) and the quantum efficiency of photocarrier generation from CT complex φwere evaluated.

| composition | *dj*_photo_/*dt* (A cm^−2^s^−1^) | *φα* + *φ*_2_*α*_2_ (cm^−1^) | *φ*_2_ (10^−2^) |
| --- | --- | --- | --- |
| 30/39.4/30/.6 | 0.165 | 0.532 | 5.06 |

**Table S4.** Summary of photoconductive parameters for better fitting.

| composition | *γ*_R_ (cm^3^ s^-1^) | *γ*_R2_ (cm^3^ s^-1^) | *γ*_T_ (cm^3^ s^-1^) | *T, M* (cm^-3^) | *β*_T_ (s^-1^) | *γ*_M_ (cm^3^ s^-1^) | *β*_M_ (s^-1^) |
| --- | --- | --- | --- | --- | --- | --- | --- |
| 30/39.4/30/.6 | 2.0×10^-13^ | 8.0×10^-12^ | 4.9 ×10^-14^ | 3.27 ×10^16^ | 85 | 1.5×10^−16^ | 0.01 |


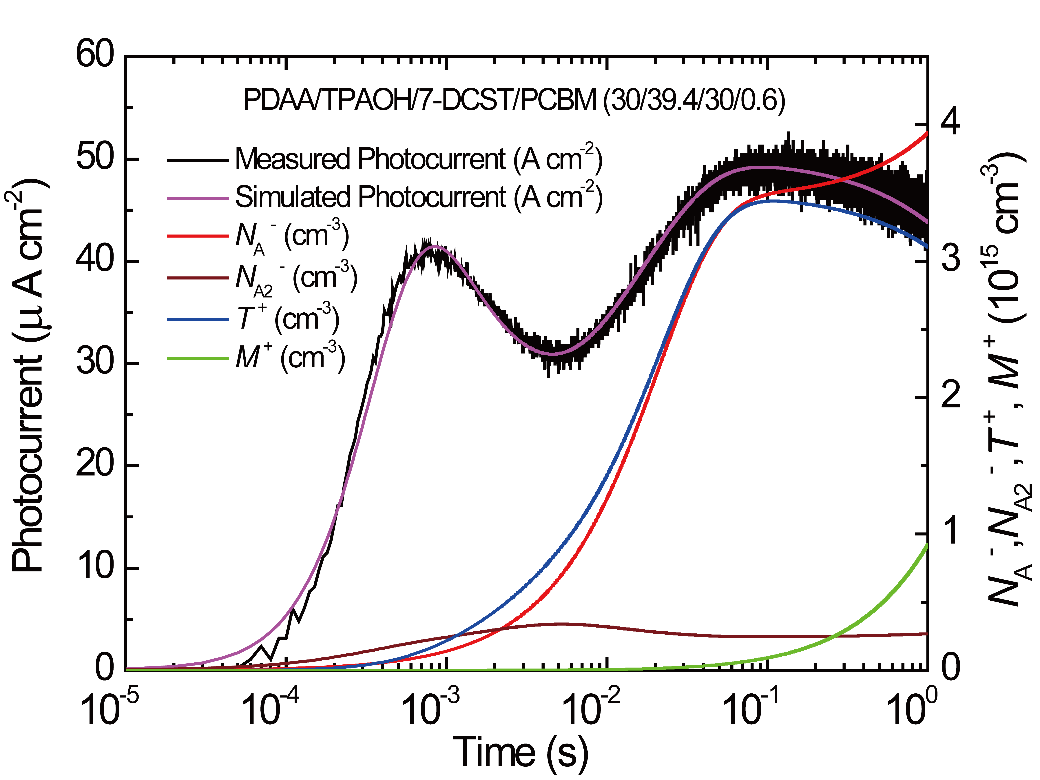


**Figure S3**. Well-fitted transient photocurrent for PDAA/TPAOH/7-DCST/PCBM (30/39.4/30/0.6) using parameters in **Tables S3** and **S4**.
